# Outcomes of ultra-high-pressure balloon angioplasty for congenital heart disease in single-center experience

**DOI:** 10.1007/s00380-025-02547-1

**Published:** 2025-05-06

**Authors:** Maiko Kondo, Yoshihiko Kurita, Yosuke Fukushima, Yusuke Shigemitsu, Kenta Hirai, Yuya Kawamoto, Mayuko Hara, Tomoyuki Kanazawa, Tatsuo Iwasaki, Yasuhiro Kotani, Shingo Kasahara, Hirokazu Tsukahara, Kenji Baba

**Affiliations:** 1 Department of Pediatrics, Okayama University Graduate School of Medicine, Dentistry and Pharmaceutical Sciences, 2-5-1, Shikata-cho, Kita-ku, Okayama-shi, Okayama 700-8558 Japan; 2 Department of Pediatrics, Okayama University Hospital, Okayama, Japan; 3 Department of Pediatric Anesthesiology, Okayama University Hospital, Okayama, Japan; 4 Department of Cardiovascular Surgery, Okayama University Hospital, Okayama, Japan

**Keywords:** Ultra-high-pressure balloon, Balloon angioplasty, Congenital heart disease

## Abstract

Angioplasty using ultra-high-pressure (UHP) balloons may successfully treat stenotic lesions refractory to high-pressure dilation. The use of UHP balloons in patients with congenital heart disease is mostly for dilation of the pulmonary artery, and there have been few reports on the effectiveness and safety of balloons for other sites. We retrospectively evaluated the efficacy and safety of the ultra-high-pressure balloon angioplasty (UHP-BA) for stenotic lesions in patients with congenital heart disease between January 2020 and December 2022 at Okayama University Hospital. A total of 78 UHP-BAs were performed in 44 patients, with a median age of 6.6 years and a median weight of 17.6 kg. The balloon types used in the UHP-BAs were Yoroi^®^ and Conquest^®^. UHP-BA performed 39 procedures for the pulmonary artery (PA), 24 for fenestration, 8 for SVC, 4 for shunt, and three for others. The lesion-specific acute procedural success rates for PA, Fontan fenestration, SVC, and shunt were 77%, 75%, 88%, and 75%, respectively. A complication of UHP-BA occurred in 3.8% (3/78). Two of the three patients had pulmonary hemorrhage, and the remaining patients had pulmonary artery embolization due to the migration of a thrombus. There were no fatal complications. Balloon dilation with UHP balloons was safe and effective not only for pulmonary artery stenotic lesions but also for SVC, Fontan fenestration, shunt, and other dilation sites in patients with congenital heart disease.

## Introduction

Transcatheter balloon angioplasty has been the treatment of choice for stenotic lesions in patients with congenital heart disease (CHD) [1–5]. Angioplasty using ultra-high-pressure (UHP) balloons may successfully treat stenotic lesions refractory to high-pressure dilation. Ultra-high-pressure balloon angioplasty (UHP-BA) was initially used to treat stenotic hemodialysis fistulas [6]. In the field of congenital heart disease, there have been only a few reports of UHP-BA treatment for primary or stent-related pulmonary artery (PA) stenosis [7–10] and almost no reports of UHP-BA treatment for other lesions, including Fontan fenestration, superior vena cava (SVC), shunt, and others. In this study, we analyzed the safety and efficacy of UHP-BA for stenotic lesions in CHD patients at our center. 

## Methods

A retrospective analysis was conducted on all patients with congenital heart disease, excluding pulmonary vein stenosis lesions, who underwent UHP-BA, defined as a rated pressure of 20 atmospheres (atm) or higher, at the Department of Pediatric Cardiology, Okayama University Hospital from January 2020 to December 2022. Cases in which UHP was used but the inflation pressure did not reach 20 atm were excluded. Pulmonary vein stenosis lesions were also excluded because many patients with pulmonary vein stenosis have pulmonary hypertension, which can be exacerbated by contrast media, and angiography is often not performed before and after UHP-BA in such cases, making data unavailable. The Research Ethics Board at Okayama University Hospital approved this study (#2408-006), and informed consent was obtained via opt-out due to the retrospective nature of this study.

The indications for UHP-BA were as follows: (1) conventional balloon angioplasty is ineffective, that is, inadequate improvement in pressure gradient or remaining waist. (2) Conventional balloon angioplasty is expected to be unsuccessful, such as when UHP-BA was performed in the previous session or has been used for similar lesions in the past.

Predicting whether conventional balloon angioplasty will be unsuccessful can be challenging. Conventional balloons, with a rated burst pressure of 10–15 atm, are superior to UHP balloons in terms of lesion accessibility. However, since their prices are almost the same in our country and excessive inflation can be avoided by stopping inflation once the waist at the stenotic site disappears, UHP balloons are prioritized when it is deemed that they can be successfully delivered.

Two types of UHP balloons, 18-Yoroi^®^ (Kaneka Corp, Osaka, Japan) and Conquest^®^ (Bard Inc, Tempe, Ariz), were used with a GM-30 Inflation Device (Nipro Corp., Osaka, Japan). Due to the difference in the compatible wire, there are two types of Yoroi^®^, 18-Yoroi^®^ and 35-Yoroi^®^. However, since only 18-Yoroi^®^ was used in this study, it will be referred to as Yoroi^®^ in this manuscript. The Yoroi^®^ (4–7 mm diameter) has rated burst pressures at 30 atm and is constructed in three layers: a polyurethane resin layer, a high-strength fiber layer, and a polyamide resin layer. The Conquest^®^ (5–12 mm diameter) has rated burst pressures ranging from 20 to 30 atm and is layered with woven ultra-high-molecular-weight polyethylene. The Yoroi^®^ and Conquest^®^ overlap in diameters ranging from 5 to 7 mm, but the balloon selection was determined by the interventionalist’s preference.

The UHP balloon size was selected based on angiography. For unstented stenotic lesions, the UHP balloon size was generally determined to be 2–3.5 times the diameter of the stenosis or up to twice the diameter of the reference vessel. A balloon with a stent diameter of − 1 to + 2 mm was selected for stent stenotic lesions in all but two cases. In one of the two exceptions, a balloon with a stent size of + 4.5 mm was selected for dilation of the stent failure, considering the diameter of the narrowest portion. In the other case, only the distal end of the stent required dilation, and a stent diameter of − 3 mm was selected to match the diameter of the peripheral vessel.

Regarding Fontan fenestration, our institution has a policy that fenestration is necessary for Fontan patients with CVP over 15 mmHg or who suffer from Fontan-related complications such as protein losing enteropathy or plastic bronchitis. The application of UHP-BA for Fontan fenestration involves enlarging an existing hole in the Fontan conduit, which is fundamentally different from procedures performed on other lesions, such as dilating stenotic segments of vessels or artificial conduits. UHP-BA for Fontan fenestration is performed relatively frequently and has, therefore, been included in this series. The size of the Fontan fenestration balloon is 4–6 mm to achieve a patency of about 4 mm.

For shunt lesions, the UHP balloon size was selected up to + 1 mm of shunt size.

Cardiac catheterizations were performed with the patients under general anesthesia or sedation. Patients were usually administered at least 100 IU/kg of heparin, maintaining activated clotting time (ACT) target range between 200 and 300 s throughout the procedure. A guide wire was introduced into the target vessel via the catheter; the balloon catheter was advanced over the guide wire. Under fluoroscopy, the balloon was inflated until the waist disappeared or until the rated burst pressure was applied, after which the balloon was rapidly deflated. After balloon angioplasty, the pressure measurement and angiograms were performed.

Medical records and angiograms were reviewed. The diameter of the stenotic lesion and the pressure gradient across the stenosis pre- and post-UHP-BA, the location of the target lesion, the type and size of the UHP balloon, and complications were evaluated. The ratio of the balloon size to the narrowest diameter was measured. In the case of the double-balloon technique, the effective balloon size was 0.82 (D1 + D2) (D1 and D2 are the diameters of the balloons used), which was calculated from the simplified Narang et al. formula from the report of Rao et al. [11, 12]. In one case of pulmonary hemorrhage complication, angiography was not performed after the balloon angioplasty, so it was not included in the calculation of the efficacy assessment. However, it is counted complication case.

As with previous reports [1–3, 7, 13], the procedure was considered successful if one of the following criteria was met: (1) an increase in vessel diameter at the site of stenosis of 50% or more. (2) A decrease in the pressure gradient across the stenosis of 50% or more.

Data are expressed as mean ± standard deviation or median (range, minimum–maximum). Continuous variables were compared using the Mann–Whitney test. Dichotomous and categorical variables were analyzed using Fisher’s exact test and Chi-square test. Probability values less than 0.05 were considered statistically significant.

## Results

During this study period, 44 patients underwent transcatheter UHP-BA. Patient characteristics and diagnosis are shown in Table 1. A total of 78 UHP-BAs were performed on 57 lesions in 44 patients, with a median age of 6.6 years (range 104 days–37.2 years) and a median weight of 17.6 kg (3.0–66.4 kg). Of the 57 lesions, 42 required one procedure, 11 required two, 3 required three, and 1 lesion required five procedures. Among 44 patients, 13 had multiple lesions. Of these, 8 had bilateral PA lesions, 2 had unilateral PA lesions and Fontan fenestration lesions, 2 had unilateral PA lesions and shunt lesions, and 1 had unilateral PA lesions and superior vena cava (SVC) lesions. Of the 78 procedures, 74 were performed using the single-balloon technique, and 4 were performed using the double-balloon technique. Regarding the balloons used, in the single-balloon technique, the Yoroi^®^ balloon was used in 51 procedures, and the Conquest^®^ balloon was used in 23 procedures. In the double-balloon technique, two procedures used two Yoroi® balloons, another used two Conquest^®^ balloons, and the remaining one used both a Yoroi^®^ and a Conquest^®^ balloon.

**Table 1:** Patient characteristics and diagnosis

Diagnosis	No. patients
Single ventricle	28
Truncus arteriosus	4
Tetralogy of Fallot	4
PA/VSD MAPCA	3
Vascular ring/sling	2
Coarctation of aorta	2
TGA	1

Ages (years)	No. angioplasty procedures
< 1	1
1–5	21
5–10	22
10–18	30
> 18	4

Body weight (kg)	No. angioplasty procedures
< 10	7
10–20	34
20–30	15
> 30	22

*PA/VSD* pulmonary atresia with ventricular septum defect, *MAPCA* major aortopulmonary collateral arteries, *TGA* transposition of the great arteries

The total success rate was 76%. The balloon-to-narrowest diameter ratio was 2.64 ± 0.69 in the success group. The balloon-to-narrowest diameter ratio was 1.92 ± 0.39 in the non-success group. The balloon-to-narrowest diameter ratio was significantly larger in the success group (p < 0.001).

The UHP-BA is for each target lesion described below (Table 2).

**Table 2:** Number of Patients, Lesions, and Procedures for Each Target Site

	PA	Fontan fenestration	SVC	Shunt	Others
No. patients	24	14	6	3	3
No. lesion	32	13	6	3	3
	RPA16, LPA16				
No. procedure	39	24	8	4	3

*PA* pulmonary artery, *SVC* superior vena cava

### UHP-BA for PA

**Fig. 1:**
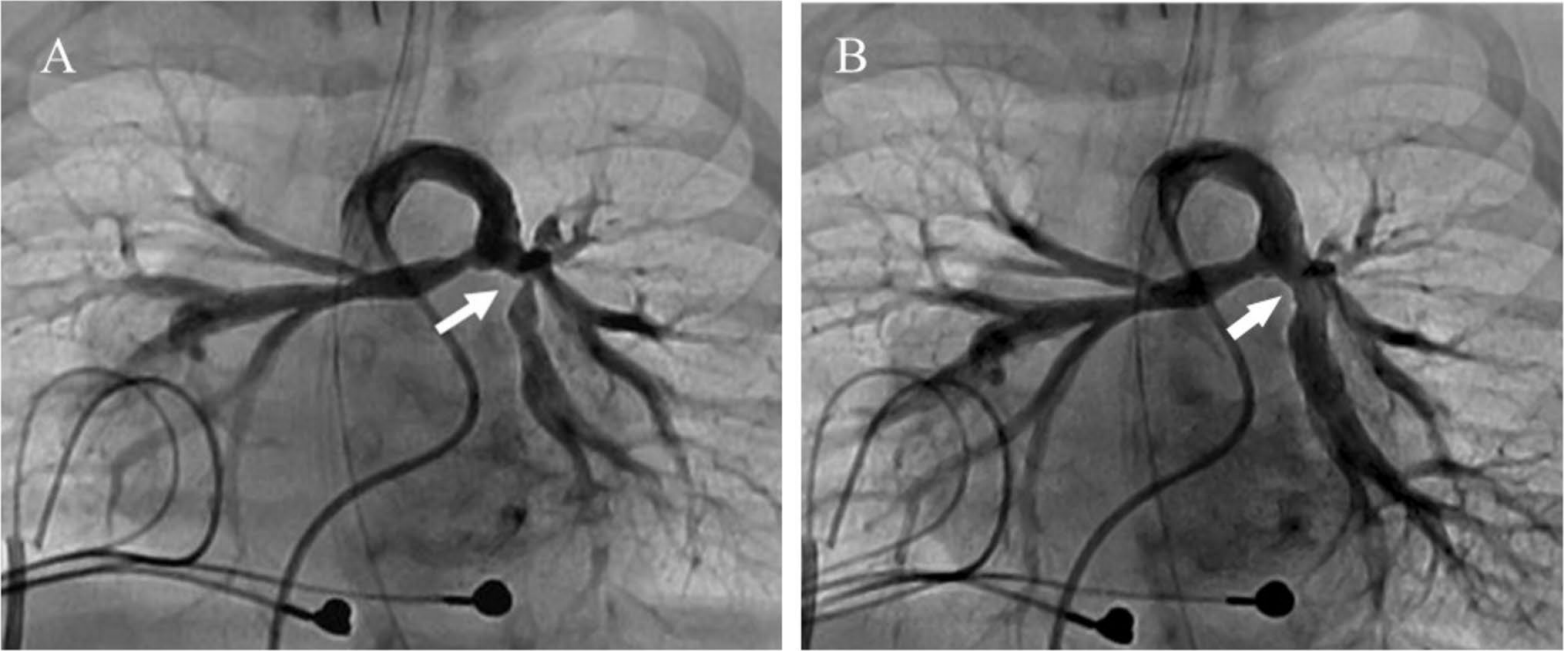
Successful balloon angioplasty of left pulmonary artery stenosis (arrow) through a right ventricle pulmonary artery shunt (RVPA shunt). Angiography of RVPA shunt before (**A**) and after (**B**) ultra-high-pressure balloon angioplasty (4 mm × 20 mm Yoroi^®^ balloon)

For PA lesions, a total of 39 UHP-BAs were performed on 32 lesions in 24 patients. The median age of the patients was 5.7 years (range 104 days–37.2 years), and the median weight was 15.7 kg (range 3.0–66.4 kg). Among the 24 patients, 16 had unilateral lesions, while 8 had bilateral lesions. Of the 32 lesions, 25 were treated with a single procedure, whereas 7 required two procedures. Of these, 3 UHP-BAs were performed at the stenting site (Fig. 1).

UHP-BA for all 39 PA lesions enlarged the stenotic lesion from 3.5 ± 1.1 mm to 5.3 ± 1.7 mm with 155 ± 26% improvement, decreased the pressure gradient across the stenotic lesion from 29 ± 24 mmHg to 23 ± 19 mmHg with 33 ± 36% improvement. The balloons used for PA were Yoroi^®^ in 20 procedures and Conquest^®^ in 16 procedures for the single-balloon technique. For the double-balloon technique, two procedures used two Yoroi^®^ balloons, and another used a combination of Yoroi^®^ and Conquest^®^ balloons. The ratio of the balloon size to the narrowest diameter was 2.35 ± 0.50.

The procedural success rate for all PA lesions is 77% (30/39). In the PA lesions, the balloon-to-narrowest diameter ratio was 2.51 ± 0.45 in the success group and 1.81 ± 0.20 in the non-success group. The balloon-to-narrowest diameter ratio was significantly larger in the success group (p < 0.001).

### UHP-BA for Fontan fenestration

**Fig. 2:**
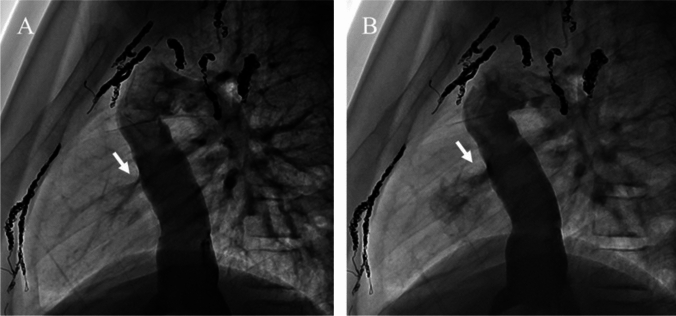
Successful balloon angioplasty of Fontan fenestration (arrow). Angiography of inferior vena cava before (**A**) and after (**B**) ultra-high-pressure balloon angioplasty (5 mm × 20 mm Yoroi^®^ balloon)

For Fontan fenestration lesions, 24 UHP-BAs were performed in 13 patients, with a median age of 12.9 years (4.7–16.5 years) and a median weight of 25.9 kg (13.1–48.4 kg). Of the 13 patients, 5 had stents implanted in the fenestration, and 8 had no stents. Of the eight patients without stents, 5 underwent one, 2 underwent two, and 1 underwent three procedures. Of the patients with implanted stents, 2 underwent one procedure, and one each underwent 2, 3, and 5 procedures. In total, 12 UHP-BAs were performed at the stenting site (Fig. 2).

UHP-BA for 24 Fontan fenestration lesions enlarged the stenotic lesion from 2.0 ± 0.6 mm to 3.7 ± 0.7 mm with 185 ± 59% improvement. Yoroi was used for all angioplasty. The ratio of the balloon size to the narrowest diameter was 2.86 ± 0.87.

The procedural success rate for all Fontan fenestration lesions is 75% (18/24). In the Fontan fenestration lesions, the balloon-to-narrowest diameter ratio was 3.13 ± 0.86 in the success group and 2.11 ± 0.25 in the non-success group. The balloon-to-narrowest diameter ratio was significantly larger in the success group (p < 0.01).

### UHP-BA for SVC

For SVC lesions, 8 UHP-BAs were performed in 6 patients, with a median age of 4.3 years (1.4–9.0 years) and a median weight of 14.8 kg (9.4–24.3 kg). Four of the six patients had stenosis at the SVC-PA anastomosis after the bidirectional Glenn, and two had stenosis at the SVC-RA junction after biventricular repair. One of these patients had stents implanted. UHP-BA for SVC lesions enlarged the stenotic lesion from 4.0 ± 1.2 mm to 6.0 ± 1.5 mm with 152 ± 18% improvement, decreased the pressure gradient across the stenotic lesion from 3 ± 2 mmHg to 2 ± 2 mmHg with 69 ± 39% improvement. The procedural success rate for SVC lesions is 88% (7/8). The balloon used for SVC was Yoroi^®^ in 1 and Conquest^®^ in 7. The ratio of the balloon size to the narrowest diameter was 2.36 ± 0.63.

### UHP-BA for shunt

For shunt lesions, 4 UHP-BAs were performed in 3 patients, with a median age of 3.5 years (2.3–5.1 years) and a median weight of 11.4 kg (6.6–13.0 kg). One of the three patients had a Blalock–Taussig shunt, one had a central aortopulmonary shunt, and one had a right ventricle-to-pulmonary artery shunt. None of these patients had stents implanted. Duration from shunt creation was 2 years (1–4.9 years). UHP-BA for shunt lesions enlarged the stenotic lesion from 2.1 ± 0.5 mm to 3.1 ± 0.4 mm with 150 ± 21% improvement. The procedural success rate for shunt lesions is 75% (3/4). Yoroi^®^ was used for all angioplasty.

### UHP-BA for other lesions

UHP-BA was performed for PA banding lesions and Fontan conduit lesions. For PA banding lesions, two UHP-BAs were performed on two lesions in two patients. Yoroi^®^ was used for all angioplasty. UHP-BA for PA banding lesions enlarged the stenotic lesion from 2.8 and 2.8 mm to 3.0 and 3.1 mm, with 107 and 111% improvement, respectively. None of these procedures were considered procedural success. The ratio of the balloon size to the narrowest diameter was 2.5.

**Fig. 3:**
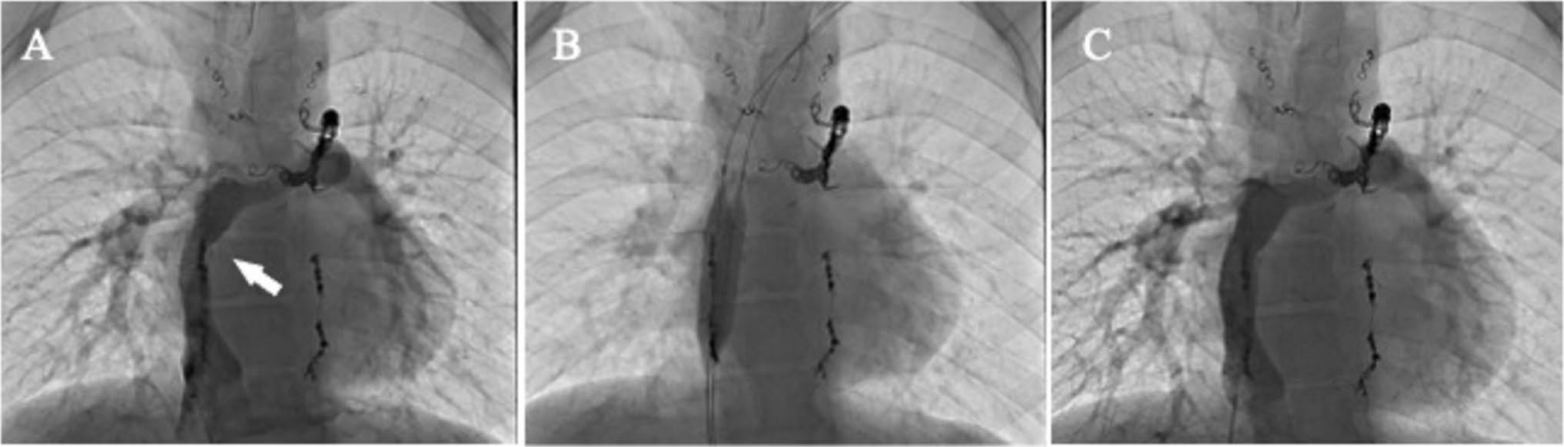
Balloon angioplasty of Fontan conduit (extra cardiac conduit with 20 mm PTFE). Angiography of inferior vena cava before (**A**) and after (**C**) ultra-high-pressure balloon angioplasty. **B** Double-balloon technique using 12 mm × 40 mm Conquest^®^ balloon and 10 mm × 40 mm Conquest^®^ balloon. Arrow: calcified conduit

For Fontan conduit lesions, only one UHP-BA was performed using the double-balloon technique by two Conquest^®^ balloons (Fig. 3). Although the stenotic lesion enlarged with 138% improvement, the pressure gradient did not reduce, and the procedure was not considered a procedural success.

### Cases of upgrading from conventional balloon to UHP balloon

Among the 78 procedures, 11 required upgrading the balloon from a conventional balloon to a UHP balloon during the same session due to residual balloon waist. The target lesions included PA (7 procedures), fenestration (3 procedures), and SVC (1 procedure). The balloon was upgraded to a UHP balloon, but its diameter was not identical to that of the previously used conventional balloon and varied across cases. In all procedures upgraded to UHP-BA, the waist successfully disappeared. The diameter of the UHP balloon without a waist was significantly larger than the waist diameter observed with the conventional balloon (*p* < 0.01). UHP-BA for 11 upgrading lesions enlarged the stenotic lesion from 2.8 ± 1.2 mm to 5.0 ± 1.7 mm with 186 ± 90% improvement. The technical success rate was 91% (10/11). The only case not considered a technical success was a right PA stenotic lesion following arterial switch operation for complete transposition of the great arteries. Balloon angioplasty was first performed using a 10-mm conventional balloon, followed by UHP-BA with a Conquest balloon of the same diameter. As a result, the narrowest lesion improved from 2.9 mm to 4.0 mm; however, the improvement rate was limited to 145%, and the pressure gradient reduction did not meet the criteria.

### Complication

A complication of UHP-BA occurred in 3 of 78 UHP-BAs (3.8%). Two of the three patients had pulmonary hemorrhage, which was caused by guidewire injury, and they were treated with mechanical ventilation using positive end-expiratory pressure and recovered with conservative treatment. The remaining patient, who had undergone UHP-BA for SVC-PA anastomosis lesion, had pulmonary artery embolization due to migration of a thrombus at the stenotic lesion and required surgical thrombectomy. There was no perioperative mortality.

### Follow-up

The median follow-up period was 3 years (range 1.6–4.5 years). During the follow-up period, additional treatment was performed on 35 of the 57 lesions. The reintervention rate was 61%. Additional balloon angioplasty (regardless of whether a UHP balloon was used) was performed on 31 lesions, while unplanned surgical intervention was selected for 4 lesions. The balloon-to-narrowest diameter ratio in patients with restenosis was 2.59 ± 0.72, while the balloon-to-narrowest diameter ratio in patients without restenosis was 2.38 ± 0.50, tending to be larger in patients with restenosis, but the difference was not significant.

## Discussion

The UHP balloon has rated burst pressure ranging from 20 to 30 atm and was initially used to treat stenotic hemodialysis fistulas [6]. The usage of UHP balloons in patients with congenital heart disease has initially been reported for stent-related PA stenosis [8, 9] and later for primary PA stenosis [7]. However, there have been almost no reports on lesions other than PA. In this study, we present evidence of the safety and efficacy of UHP-BA in CHD patients with PA stenotic lesions as well as non-PA stenotic lesions such as SVC, Fontan fenestration, shunt, and others.

The Yoroi^®^ has a three-layer design consisting of a polyurethane resin layer, a high-strength fiber layer, and a polyamide resin layer, and the Conquest^®^ has a layered structure with woven ultra-high-molecular-weight polyethylene. Due to the cross-layered material on the surface that does not stretch even when inflated under higher pressure, the UHP balloon is non-compliant. The UHP-BA is effective not only because of its ability to apply higher pressure but also because its non-compliant nature prevents balloon shape deformation and slippage, making it easier to stabilize the balloon position during inflation. The non-compliant nature of the UHP balloon also reduces the risk of vascular injury because it prevents overexpansion or so-called “dog-boning” due to excessive wall stress on the vessel adjacent to the stenotic lesion, which would occur when using a more compliant balloon.

Selecting the proper balloon size is even more critical to reduce the risk of vascular injury and increase the effectiveness of the UHP-BA. Previous reports using standard or high-pressure balloons recommend a balloon size of 2–5 times the diameter of the stenosis [2–4]. Using the UHP balloon for stented PA stenosis lesions, one reported a smaller balloon size of 1.2–1.3 times the diameter of the stenosis [8], while another reported that 2.57 times was the threshold for successful or unsuccessful procedures, with an average of 3.9 times for successful procedures [7]. In our PA stenosis lesions, the ratio of the balloon size to the narrowest diameter was 2.35 ± 0.50, smaller than 2.57 but within the reported acceptable range. Our UHP balloon size selection was similar for PA stenotic lesions as well as other lesions: 2–3.5 times the diameter of the stenosis or up to 2 times the reference vessel diameter for unstented stenotic lesions, and − 1 to + 2 mm of stent diameter for stented lesions fundamentally.

In this study, the total acute success rate of UHP-BA in congenital heart disease was 76% (59/78). The lesion-specific acute procedure success rates for PA, Fontan fenestration, SVC, shunt, and others were 77% (30/39), 75% (18/24), 88% (7/8), 75% (3/4), and 0% (0/3), respectively. In previous reports, the success rate for PA lesions was 78.4% [7] with UHP balloons and 50–64% [1, 2, 14, 15] with conventional standard or high-pressure balloons. Moreover, success rates for balloon dilation for SVC and shunt have been reported as 71% [16] and 84.8–91% [17–19], respectively. Compared to previous reports, the success rate is considered satisfactory.

This study does not compare cases treated with a UHP balloon to those treated with a conventional balloon, making it difficult to demonstrate the superiority of UHP-BA over conventional balloon angioplasty. However, in this study, among 11 cases where UHP balloons were upgraded following insufficient efficacy of conventional balloons, all conventional balloon waists disappeared, and the vessel diameter was significantly increased. This demonstrates the utility of UHP-BA.

Reintervention was performed in 32 lesions (56%) during our median follow-up period of 3 years. The restenosis rate with conventional balloon dilatation for pulmonary artery stenosis was estimated to be 10–44% [1, 2, 15, 20–22], and the use of a UHP balloon did not reduce this restenosis rate [7]. This study’s reintervention rate might be higher than the previous restenosis rate, which was thought to be due to this study included not only the pulmonary artery but also other lesions, as well as aggressive balloon dilation to accommodate growth.

Applying higher pressure with the UHP balloon increased the acute success rate but did not reduce the rate of subsequent restenosis. A larger balloon-to-waist ratio was reported to be associated with the risk of restenosis. In our data, the ratio of balloon-to-narrowest diameter in patients with restenosis tended to be greater than in those without restenosis, but the difference was not statistically significant.

In this study, Yoroi^®^ (4–7 mm diameter) and Conquest^®^ (5–12 mm diameter) were used, but the use of the larger Atlas (Bard Inc, Tempe, Ariz) (12–26 mm diameter) has been reported [8]. However, UHP balloons larger than 12 mm in diameter, including the Atlas, are unavailable in Japan as of the end of this study period. When a UHP-BA larger than 12 mm is required, we must perform a double-balloon technique, which was conducted in 4 cases in this series. It is gratifying that, after this study period, the Atlas Gold balloon became available in Japan as of September 2024, albeit with a maximum diameter of 16 mm.

In our series, there were no fatal complications or vessel ruptures, even with the UHP balloon. The complication rate was 3.8% (3/78), comparable or lower than the conventional balloon series. Pulmonary hemorrhage, which was caused by guidewire injury, occurred in 2 patients, but all patients improved with conservative treatment using positive pressure ventilatory management. Pulmonary artery embolization resulting from UHP-BA for SVC-PA anastomosis lesions was caused by migration of thrombus in the stenotic lesion prior to treatment, which was not due to the UHP-BA and is considered a limitation of balloon therapy. UHP-BA is considered a safe treatment with proper size selection.

## Study limitations

This study is a retrospective, non-randomized review of a small cohort from a single institution and thus suffers from limitations intrinsic to those boundaries. One of the criteria for indication is “conventional balloon angioplasty is expected to be unsuccessful.” However, since this can sometimes be difficult to predict, cases with unclear indications may have been included. The follow-up period of this cohort is relatively short, so further follow-up studies are necessary.

## Future directions

Japan has a database called the Japanese Society of Congenital Interventional Cardiology (JCIC) registry, which records catheter interventions from most institutions nationwide. Utilizing this registry effectively, we aim to advance a multicenter collaborative study on UHP-BA.

## Conclusion

Balloon dilation with UHP balloons achieved a total success rate of 76% was effective not only for pulmonary artery stenotic lesions but also for SVC, Fontan fenestration, and shunts in patients with congenital heart disease. The complication rate associated with balloon dilation is low (3.8%), and dilation of stenotic lesions in patients with congenital heart disease using UHP balloons is considered a useful treatment option.
